# A Tale of Two “Forests”: Random Forest Machine Learning Aids Tropical Forest Carbon Mapping

**DOI:** 10.1371/journal.pone.0085993

**Published:** 2014-01-28

**Authors:** Joseph Mascaro, Gregory P. Asner, David E. Knapp, Ty Kennedy-Bowdoin, Roberta E. Martin, Christopher Anderson, Mark Higgins, K. Dana Chadwick

**Affiliations:** Department of Global Ecology, Carnegie Institution for Science, Stanford, California, United States of America; DOE Pacific Northwest National Laboratory, United States of America

## Abstract

Accurate and spatially-explicit maps of tropical forest carbon stocks are needed to implement carbon offset mechanisms such as REDD+ (Reduced Deforestation and Degradation Plus). The Random Forest machine learning algorithm may aid carbon mapping applications using remotely-sensed data. However, Random Forest has never been compared to traditional and potentially more reliable techniques such as regionally stratified sampling and upscaling, and it has rarely been employed with spatial data. Here, we evaluated the performance of Random Forest in upscaling airborne LiDAR (Light Detection and Ranging)-based carbon estimates compared to the stratification approach over a 16-million hectare focal area of the Western Amazon. We considered two runs of Random Forest, both with and without spatial contextual modeling by including—in the latter case—x, and y position directly in the model. In each case, we set aside 8 million hectares (i.e., half of the focal area) for validation; this rigorous test of Random Forest went above and beyond the internal validation normally compiled by the algorithm (i.e., called “out-of-bag”), which proved insufficient for this spatial application. In this heterogeneous region of Northern Peru, the model with spatial context was the best preforming run of Random Forest, and explained 59% of LiDAR-based carbon estimates within the validation area, compared to 37% for stratification or 43% by Random Forest without spatial context. With the 60% improvement in explained variation, RMSE against validation LiDAR samples improved from 33 to 26 Mg C ha^−1^ when using Random Forest with spatial context. Our results suggest that spatial context should be considered when using Random Forest, and that doing so may result in substantially improved carbon stock modeling for purposes of climate change mitigation.

## Introduction

Machine learning algorithms are increasingly being applied in image analysis problems ranging from face recognition [1] to self-driving vehicles [2]. Recently, the Random Forest algorithm [3], has been used in global tropical forest carbon mapping [4]. However, there is considerable resistance to the use of machine learning algorithms in ecological applications, as the discipline has been the purview of traditional parametric statistics for decades [5], [6]. The cause for concern is genuine: Random Forest has not often been applied to spatial mapping applications, and there has been limited evaluation of its performance in such applications relative to alternative and more traditional methods. Here we present a side-by-side comparison of Random Forest-based carbon mapping predictions relative to the reliable and often-used approach of stratification-based sampling [7].

The problem of tropical forest carbon mapping continues to challenge ecologists and remote sensing experts. In practice, measuring the amount of carbon stored in a patch of forest is straightforward, if logistically challenging. Plant biomass may be harvested, dried and weighed [8], and from this material the carbon fraction determined [9]. However, it is easy to see that such efforts would be futile for determining spatially explicit carbon stock estimates at larger scales. Traditional field campaigns utilize national forest inventory networks—grids of field plots within which tree diameters, heights and wood densities are measured, and allometric models to relate such measurements to estimated carbon stock per tree [10]. But while such networks may be sufficient for estimating total carbon stock in a habitat type, ecoregion or jurisdiction, they are inadequate for estimating spatially explicit carbon stocks. Even immediately adjacent to a particular field plot, an investigator or landowner has much lower predictive power to estimate carbon stock compared to their ability to predict regional totals. Yet, such spatially-explicit carbon estimates are essential for many ecological applications as well as for carbon emissions programs such as the United Nations' Reduced Emissions from Deforestation and Forest Degradation (REDD+) effort [11].

Remote sensing technologies—and particularly LiDAR (Light Detection and Ranging)—have been used to estimate spatial variation in carbon stocks [4], [12]–[16]. Whether from airborne or spaceborne platforms, laser scanning technologies can measure aspects of forest structure that are similar to those measured in field plots. For instance, tree height is determined often more accurately with LiDAR than from the ground via traditional techniques such as clinometer trigonometry, particularly in dense, tall-statured tropical forests. Still, while LiDAR measurements offer a possible spatial mapping tool for carbon estimates, they too reach a geographic limit due to cost and logistical considerations [17]. Aircraft cannot yet cover all the world's tropical forests, and spacecraft are limited to a net-like sampling scheme [4], [15]. Thus, additional data from satellite inputs, such as Landsat, Shuttle Radar Topography Mission (SRTM), Tropical Rainfall Mapping Mission (TRMM), Moderate Resolution Imaging System (MODIS) and other sources are used to scale up LiDAR-based carbon estimates [17].

Various regional, jurisdictional and global tropical forest carbon mapping approaches that have utilized LiDAR measurements as the principal carbon estimator have employed several different techniques to extend or scale up their LiDAR-based carbon estimates [4], [7], [14], [15], [17]–[20]. Stratification is most often employed [7]; this method involves identifying unique classes often with a vegetation map [18], or by isolating unique combinations of input variables into distinct classes similar to a vegetation map [17], [20]. Random Forest has been used in few carbon (or biomass) mapping efforts to date [4], [16], [21]. Random Forest is a machine-learning algorithm that fits multiple decision trees to input data using a random subset of the input variables for each tree constructed; the mode of these trees is used to create an “ensemble” tree that is used for prediction. Random Forest has a large potential upside: it is non-parametric, insensitive to data skew, robust to a high number of variable inputs, and the algorithm purportedly “cannot” overfit [5]. However, these purported benefits—particularly the lack of overfit—have not been tested using spatial data. Ecological applications of Random Forest are increasing [22], [23], but skepticism about the method remains.

Here we evaluate the performance of Random Forest as a spatial upscaling tool for LiDAR-based carbon sampling, and we compare its performance to the more traditional stratification approach. We utilize a focal study area of 16 million hectares of tropical forest, swamp, and used lands within the Marañon and Ucayali watersheds in Northern Peru. The area harbors enormous ecological heterogeneity, including lowland *terra firme* and floodplain forests, swamps, marshes, mid- and high-elevation forests, and heavily utilized lands within each of these habitat types, including pasture, mining, oil extraction and selective logging.

## Methods

### Study area

**Figure 1:**
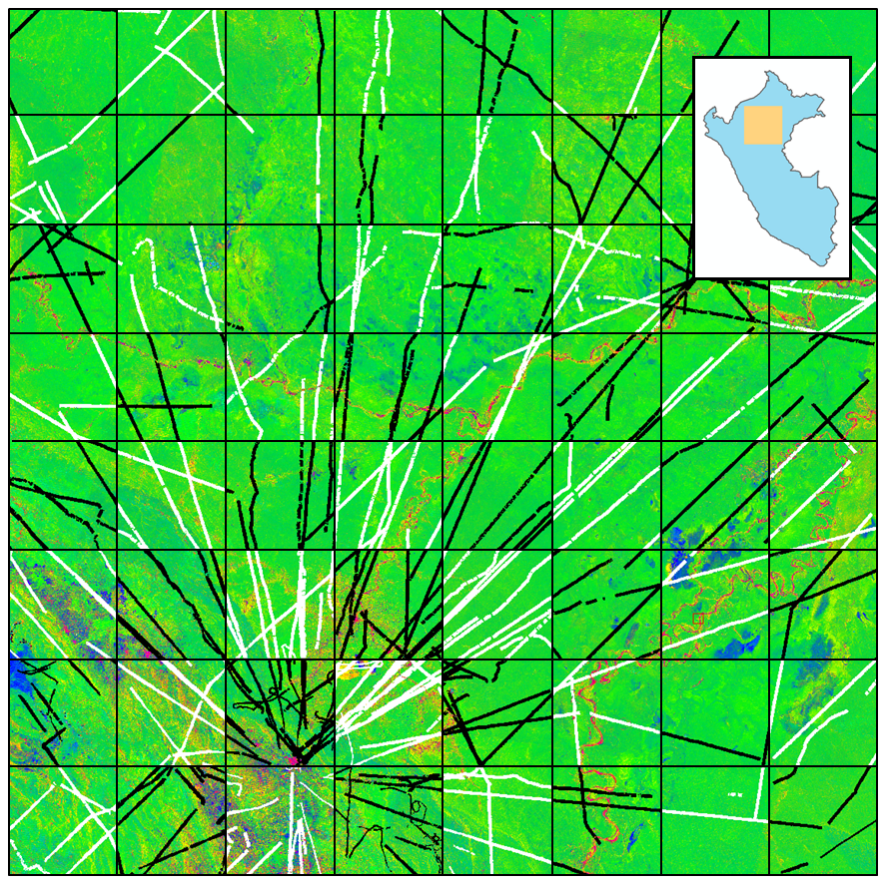
Fractional cover of photosynthetic vegetation (PV; green), non-photosynthetic vegetation (NPV; blue), and bare substrate (S; red) of our focal study region in Northern Peru. The inset shows the location of the focal area within Peru. The region spans 16 million ha of ecological heterogeneity within the Marañon and Ucayali Watersheds. For carbon modeling purposes, airborne LiDAR data from CAO were divided using a checkerboard configuration, with 694,243 ha of calibration data (white) and 669,943 ha of validation data (black).

The focal area for this study is a 16 million ha region spanning from a SW corner of 77.557° West Longitude 6.962° South Latitude to a NE corner of 73.942 West Longitude 3.349° South Latitude (Figure 1). The focal area covers an ecoregion of enormous biophysical and floristic variation, and is among the most biologically diverse regions in Amazonia [24]. Ground elevations range from 90 m a.s.l. in the eastern section of the focal area to 3884 m in the southwestern portion. The area is dissected by a series of rivers draining into the upper Amazon, including the Nanay, Tigre, Marañon, Pacaya, Samiria, and Ucayali rivers. The Pacaya-Samiria National Reserve found near the center of the focal area is a swampland covering more than 20,000 km^2^. To the east of the swamp, upland and rolling *terra firme* soils extending towards Brazil contain very high biomass stocks, and to the west of the swamp, the Pastaza Fan, Nauta and Pebas formations harbor a wide array of forest compositions and structures [25]–[27].

### LiDAR data

The LiDAR data were collected using the Carnegie Airborne Observatory (CAO) Airborne Taxonomic Mapping System (AToMS) [28]. The AToMS scanning LiDAR sensor is full waveform, but the work presented here relied only on the discrete return data of up to four returns per pulse in order to make the results applicable to a much wider range of LiDARs currently in operation throughout the world [29]. AToMS LiDAR was operated at 2,000 m above ground level with 1.12 m spot spacing, a 30° field of view, and a pulse repetition frequency of 50 kHz, for which the aircraft maintained a ground speed of ≤110 knots. Laser beam divergence is customized to 0.56 mrad (1/e).

Although carbon estimation can be accomplished with many LiDAR metrics, we relied on a simple metric of “top-of-canopy height” (TCH) for this study. TCH, estimated in m, is determined in two steps: (1) ground and surface models are generated from the 1.12 m discrete return 3-D LiDAR point cloud data collected by CAO, (2) the ground model is subtracted from the surface model to produce TCH.

### Carbon estimation

The focus of this study was to quantitatively compare two approaches—stratification and Random Forest—for scaling up airborne LiDAR-based estimates of forest carbon density to larger regional areas beyond the LiDAR coverage. For purposes of carbon mapping, the field calibration to LiDAR data is also critically important, but we did not evaluate it in this study. Asner and Mascaro [30] present a database of carbon calibration plots, and from these we subset 214 plots for Peruvian forests (ranging from 0.28 ha to 1.0 ha in size). The following equation was determined using maximum likelihood: ACD = 0.3124*TCH* ^1.854^, where TCH is the top of canopy height (m), and ACD is aboveground carbon density of all stems ≥10 cm in diameter (Mg C ha^−1^). This equation yields an R^2^ of 0.84 and RMSE of 26 Mg C ha^−1^ (see also [29]). The units of carbon estimated by this equation should be viewed for the purposes of this study as a consistent and reasonable output upon which to base the upscaling analyses for the region. Importantly, we emphasize that the present study is very unlikely to be influenced by the LiDAR calibration model used; Random Forest is a non-parametric algorithm, and any changes to the LiDAR-ACD calibration model would have a minimal impact on the magnitude of the ACD values and an even lower impact on the spread of those values.

### Large-area data inputs

**Figure 2:**
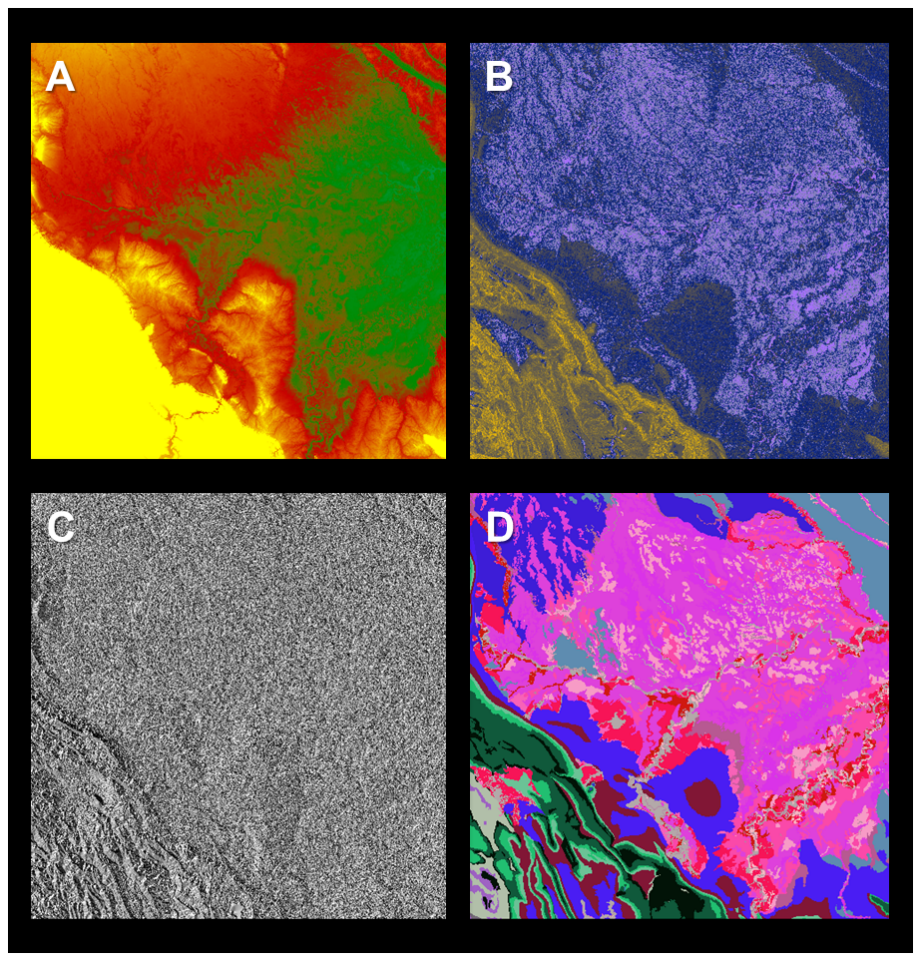
In addition to the fractional cover map shown in Figure 1, four additional maps were created as input to the stratification and Random Forest models. (a) SRTM elevation ranging from a low of 90 m a.s.l. (green) to a high of 3884 m a.s.l. (yellow), (b) SRTM slope ranging from level inundated areas (light purple) to steep cliffs and rock faces (yellow), (c) SRTM aspect ranging from a bearing of zero degrees (black) to just under 360 degrees (white), and (d) habitat type, with broad variation highlighted by kaleidoscopic color. In addition, the second of two Random Forest models included four axes of position information (see Methods).

An overview of each large-scale input variables is provided in Table 1 and described in detail here; see also Figures 1, 2. Each data layer was prepared for Peru in its entirety, from which the focal area was subset.

**Table 1:** Variables used to support three alternative upscaling methods for LiDAR-estimated tropical forest carbon stocks in a 16 million ha focal region in Northern Peru.

		*Upscaling method*		
Input Variable	Explanation	Stratification	Random Forest	Random Forest with position information
easting	UTM X coordinate			X
northing	UTM Y coordinate			X
diagx	X coordinate after 45 degree clockwise image rotation			X
diagy	Y coordinate after 45 degree clockwise image rotation			X
frac_soil	Percent cover of soil as determined by Landsat image processing with CLASlite (%)			X
frac_pv	Percent cover of photosynthetic vegetation as determined by Landsat image processing with CLASlite (%)	X	X	X
frac_npv	Percent cover of non-photosynthetic vegeation as determined by Landsat image processing with CLASlite (%)	X	X	X
elevation	SRTM elevation above sea level (m)	X	X	X
slope	SRTM slope (degrees)	X	X	X
aspect	SRTM aspect (degrees)	X	X	X
geoeco	Habitat class as determined by synthetic integration of national geological map, NatureServe and other sources	X	X	X

First, we mosaicked ten 90-m resolution Shuttle Radar Topography Mission (SRTM) tiles [31] to produce a baseline elevation map of the focal area in Peru (Figure 2). The original data were resampled to 100-m resolution using pixel averaging, and from these data we produced topographic models of slope and aspect using a 3×3 sliding kernel (i.e., the slope or aspect of the center pixel is calculated based on the elevations of all 9 pixels in the local environment of the center pixel).

Second, we processed 1071 Landsat 5 and Landsat 7 scenes (SLC off) taken in 2011 using Carnegie Landsat Analysis System lite (CLASlite). CLASlite automates radiometric correction and uses Monte Carlo Unmixing (MCU) to produce estimates of the percentage cover of soil, photosynthetic vegetation (PV), and non-photosynthetic vegetation (NPV) in every image pixel [32]. We used a pixel-selection algorithm (median Normalized Difference Vegetation Index, NDVI) to produce a “best-pixel” 2011 mosaic of 30 m-resolution MCU fractional cover. Due to persistent clouds in several regions, especially mid- and high-elevation forests, the resulting Landsat MCU mosaic lacked coverage for 2.4% of the area. To plug these gaps, we mosaicked our Landsat fractional cover mosaic overtop of 500-m MCU output from MODIS. In this case, all MODIS fractional cover estimates were normalized with co-occurring Landsat pixels prior to mosaicking.

Finally, we incorporated a 134-class habitat map to represent geological and soil variation thought to regulate forest properties in Amazonian Peru and nearby regions [25], [26], [27], [33]. The base input for this map was a national geological map for Peru to provide information on geologic and edaphic patterns in the study area [34]. Due to the importance of recent Quaternary fluvial features that were not included in the base map, we supplemented it with information from the NatureServe national vegetation map [35]. Lastly, we manually edited the geological map to account for recent findings on edaphic and floristic patterns in the region [25], [33].

### Upscaling methodology

We aligned all layers of the input data and resampled to 1-ha resolution using nearest neighbor resampling. We aligned corresponding 1.12-m resolution CAO LiDAR TCH data and determined average TCH in each 1-ha grid cell (follows [30]). From the large extent of the input data, we performed all upscaling on an area bounding the entire Marañon Watershed to avoid edge effects for our focal area of 16 million hectares.

#### Stratification

We stratified the input variables (Figures 1–2) according to quantiles. Our goal was to produce as many unique (and useful) classes of habitat variation in order to map carbon variation among these classes [7]; however, an inordinately high number of breaks among the various input variables quickly results in too high a number of classes; ideally most classes will maintain a LiDAR sample of more than 100 ha or 1% [17], [20], but in this case sampling was very dense (overall 8.5% of the focal area). We dispersed 20 bins non-randomly among our continuous input variables (i.e., STRM elevation, slope and aspect; CLASlite MCU soil, PV and NPV), based on the strength of those variables in influencing carbon stocks (Table 2). In previous studies, for example, PV and elevation were found to be the primary controlling variables on carbon stock variation in Amazonian forests (follows [17]). We subsequently intersected all unique variable combinations with the 134-class habitat map. The resulting classified map contained 8,035 classes. We intersected this class map with the CAO LiDAR data (for all of the Marañon region and surrounding environs, to avoid edge effects) to estimate median LiDAR-carbon content for each class; this median value was then mapped onto all pixels within that class using the class map.

**Table 2:** Bin ranges for input variables used to produce a stratified map of the region over which carbon modeling was performed (see methods).

Soil	PV	NPV	Elevation	Slope	Aspect
[0, 5)	[0, 85)	[0, 6)	[0, 136)	[0, 1.5)	[0, Inf)
[5, Inf)	[85, 88)	[6, 13)	[136, 193)	[1.5, Inf)	
	[88, 90)	[13, Inf)	[193, 443)		
	[90, 91)		[443, Inf)		
	[91, 92)				
	[92, 93)				
	[93, 94)				
	[94, Inf)				

Twenty total bands were dispersed non-randomly according to the strength of each variable in predicting carbon stocks, which has been shown to be an effective stratification method in previous studies (e.g., [17]). Thereafter, the input variables were subset by quantiles to determine bin ranges for the bands. These class combinations were subsequently intersected with a 134-class habitat map as described in the methods, resulting in 8,035 unique classes within the focal area of the present study. A hard bracket indicates values “greater than or equal to”, while a parenthesis indicates values that are “less than”.

#### Random Forest

We utilized the Random Forest algorithm (as contained within the R package “randomForest” version 4.6–7; R version 2.15.2 [36]) to produce a mapping prediction from the same input data layers as used in stratification. Although the Random Forest algorithm tested does include a built-in “out-of-bag” validation scheme, the effectiveness of this internal metric has not been tested with spatial data to our knowledge. Thus, to rigorously test Random Forest, we limited the model input data to a systematic subset based on a 50-km (on a side) grid cell (Figure 1). This limitation required the model to predict across large validation regions containing no input data. Combined, the focal region contained 36 calibration cells and 36 validation cells, providing a 50% leave out strategy—extremely conservative compared to most spatial modeling techniques (see, e.g., [37]).

We considered two separate “runs” of Random Forest, each using an identical set of 80,000 randomly selected input pixels among the calibration cells, which was at the limit of our computational resources. First, we produced a Random Forest model based on the large-scale input variables alone. Second, we produced a Random Forest model based on the input variables plus an additional four “position” parameters: x and y coordinates, combined with two diagonal coordinates (i.e., columns and rows for the image stack running from NW-to-SE and SW-to-NE, respectively). Position information can be critical in modeling underlying geographic trends within ecological data [38], [39], and is now used in many examples of spatial modeling for purposes of predicting ecological trends [40]–[42]. We did not include such variables to imply any mechanistic control, but rather to serve the applied goal of accurately predicting carbon stock variation. We refer to this run of Random Forest throughout as that having “position information”.

### Model comparison and evaluation

**Figure 3:**
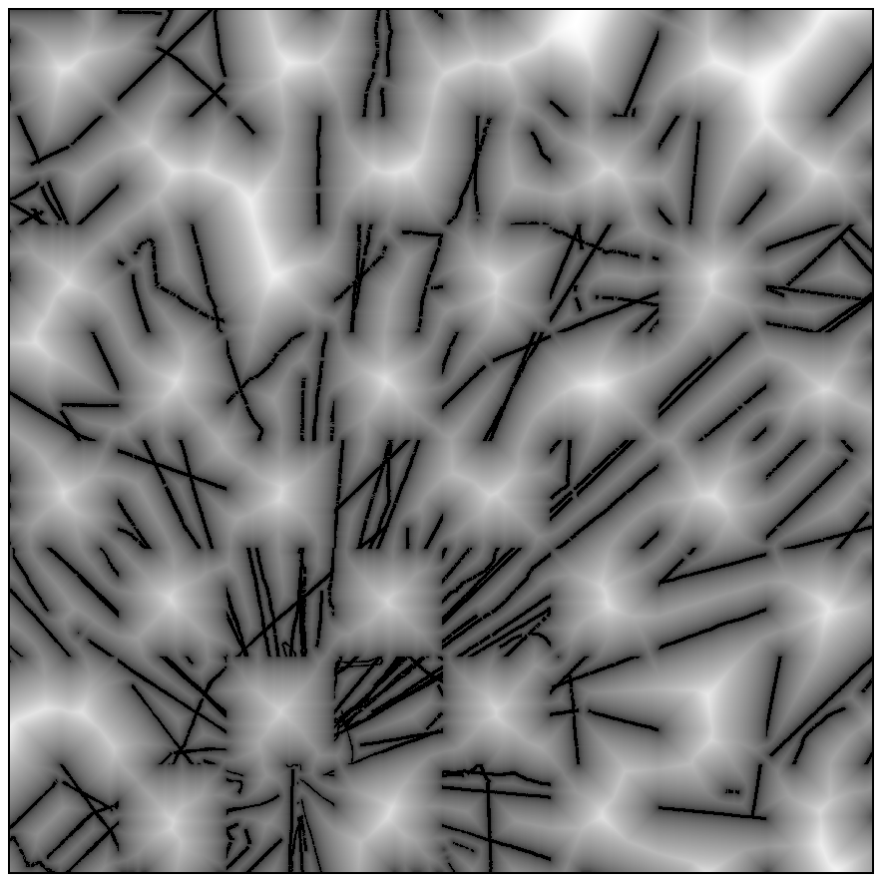
A distance transform map of the LiDAR-based carbon density calibration data used to evaluate the performance of the stratification and Random Forest models with increasing distance from aircraft observations. White areas indicate the greater distance from the calibration LiDAR flightlines in black.

We examined the resulting carbon maps side-by-side, first by considering differences among the maps. We then assessed performance by comparing the predicted ACD values within the 36 validation cells against LiDAR-observed ACD within those same cells. We used a distance transform of the extent of CAO calibration input data to determine whether the model performances were affected by increasing distance from CAO sample LiDAR data (Figure 3). The distance transform employed an approximate Euclidian distance algorithm which reproduces Euclidian distance effectively but is less computationally intensive—specifically “morph distance” in IDL (see also [43]).

We also considered the spatial autocorrelation of each model's residuals using two approaches. First, we created omnidirectional semivariograms (R package “geoR”, version 1.7–3 [36]), which depict the correlation of observations according to increasing distance between those observations. Due to computational limitations, we created semivariograms using a random subset of 15,000 residuals (i.e., consistent spatially across all three upscaling approaches). Second, we applied Moran's I, an index of spatial autocorrelation (R package “ape”, version 3.0–8 [36]); Moran's I ranges from −1 to 1, where positive values indicate clumping and negative values indicate organized opposition (i.e., a chessboard pattern). Due to computational limitations, we assessed Moran's I within a randomized subset of 5,000 observations (i.e., consistent spatially across upscaling approaches).

Finally, we considered over-fitting by comparing the internal “out-of-bag” percent variation explained reported by the Random Forest algorithm to the percent variation explained in the 36 validation cells left out of the model input data.

## Results

**Figure 4:**
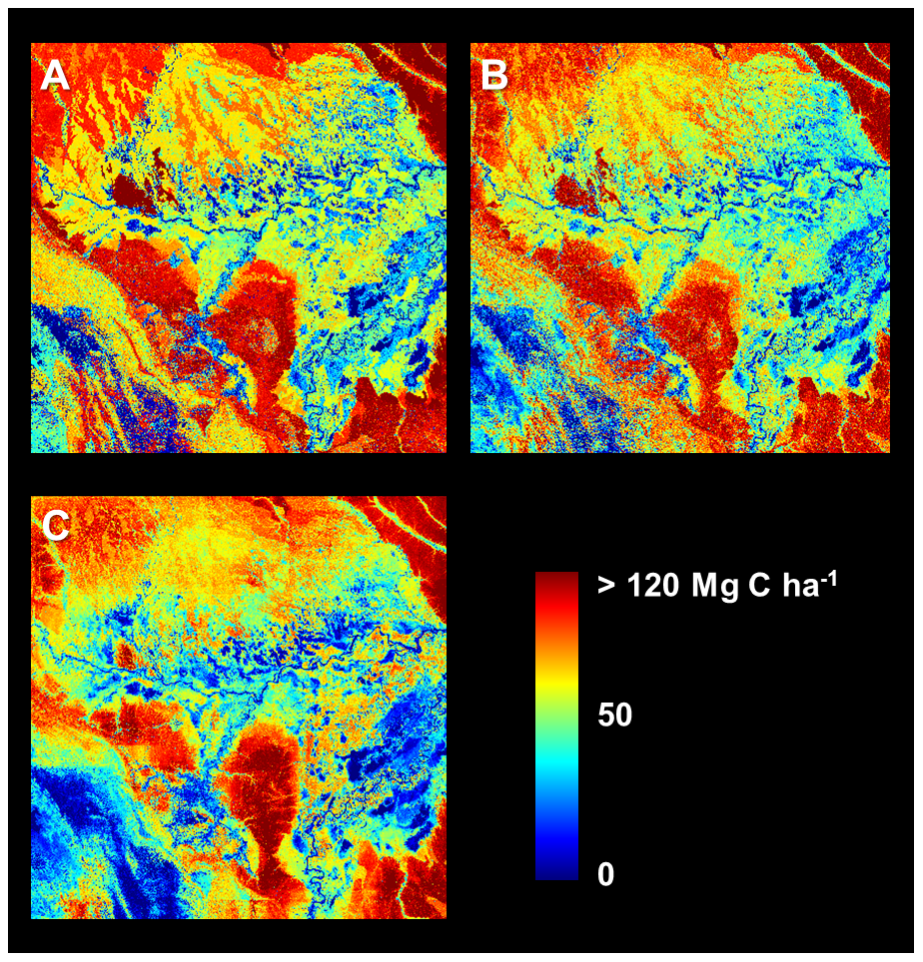
Predicted carbon stocks using three different methodologies. (a) Stratification and mapping of median carbon stocks in each class, (b) Random Forest without the inclusion of position information, (c) Random Forest using additional model inputs for position.

**Figure 5:**
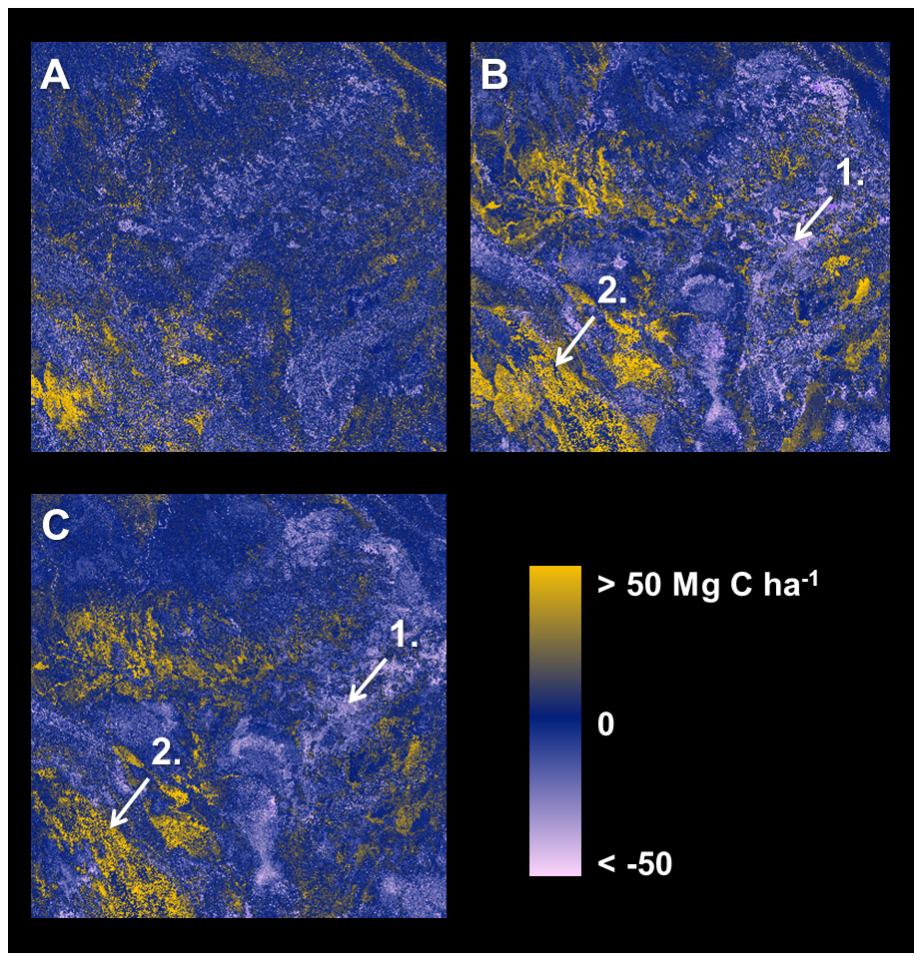
Three side-by-side carbon map comparisons. (a) Stratification minus Random Forest without position information, (b) Stratification minus Random Forest with position information, (c) Random Forest without position information minus Random Forest with position information. Areas consistently lower when position information is included (yellows in b and c) are largely low, inundated swamps and wetlands or mid-elevation pasturelands—all of which maintain high levels of photosynthetic vegetation cover (PV) but are comprised of lower carbon stocks in the airborne LiDAR data. Light blue areas (in b and c) are mostly low-elevation floodplain forests. See Discussion section regarding two annotated regions.

Stratification and Random Forest (both with and without position information) yielded predicted maps of ecosystem carbon stock that highlighted enormous variation across the 16 million-hectare focal area (Figure 4). The Pacaya-Samiria Swamp (center and east of the region) was generally modeled as containing low carbon stocks ranging from near 0 to ∼50 Mg C ha^−1^, but also showing considerable heterogeneity throughout the swampland. Upland *terra firme* forests were modeled consistently among the three approaches, including in the NE, SE, and NW corners of the focal area. However, several regions exhibited pronounced differences when using Random Forest with position information relative either to the other two approaches (Figure 5).

**Figure 6:**
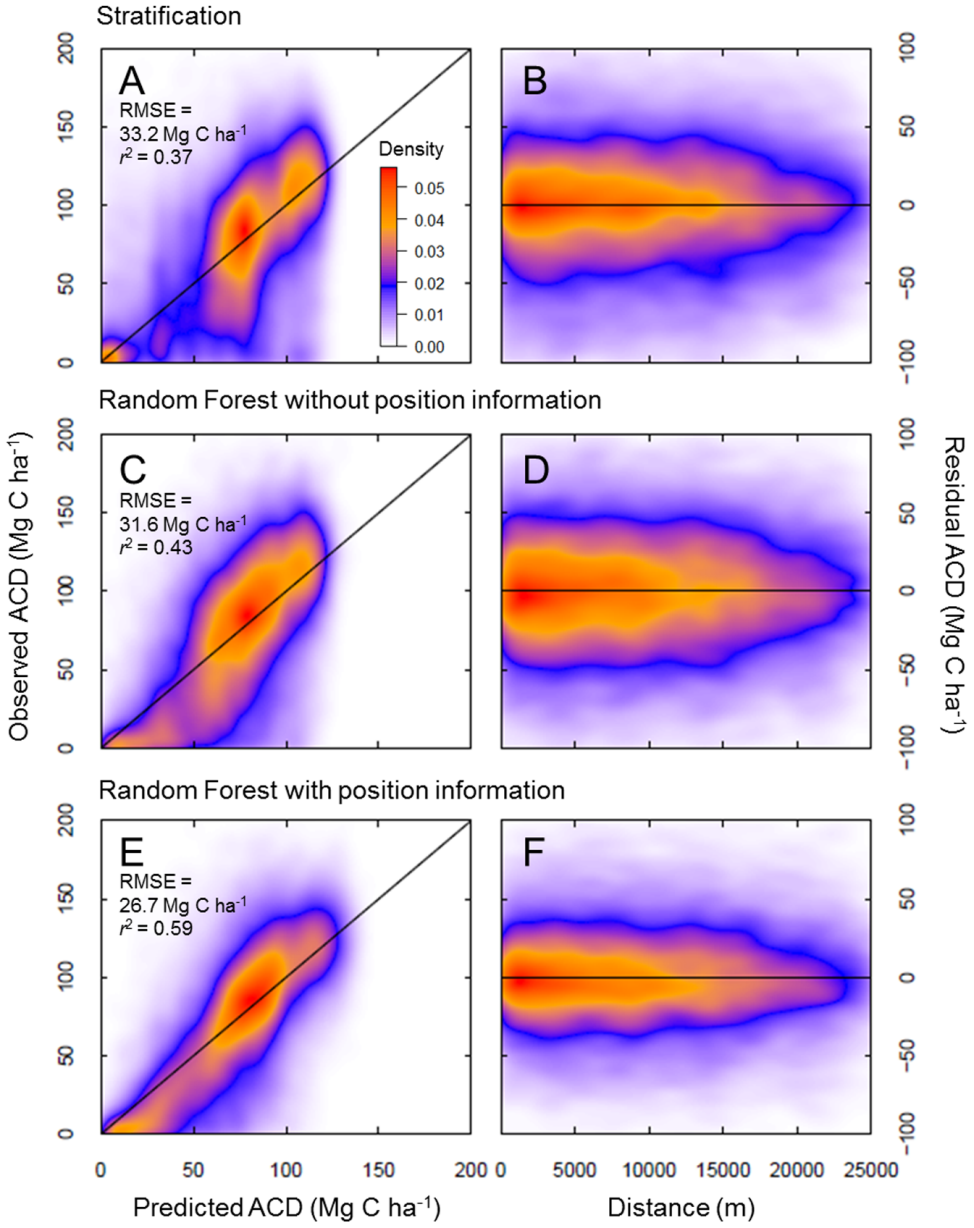
Performance of three modeling techniques as assessed in 36 validation cells. Left panels highlight model performance against LiDAR-observed aboveground carbon density from CAO aircraft data (Mg C ha^−1^), while right panels highlight the model performance by increasing distance from CAO aircraft data. The color-scale reflects the two-dimensional density of observations, adjusted to one dimension using a square root transformation.

However, a clear benefit of using Random Forest with position information emerged during statistical comparison of the approaches (Figure 6). As a baseline, stratification yielded a RMSE of 33.2 Mg C ha^−1^ and adjusted r^2^ of 0.37 (predicted versus observed). A modest improvement was detected with Random Forest without position information (RMSE = 31.6 Mg C ha^−1^, adjusted r^2^ = 0.43), but the Random Forest model that included position information yielded a 20% improvement in RMSE (26.7 Mg C ha^−1^) and a 60% improvement in adjusted r^2^ (0.59). The improvement when using Random Forest with position information appeared to be consistent at all distances from CAO LiDAR data (Figure 6).

**Figure 7:**
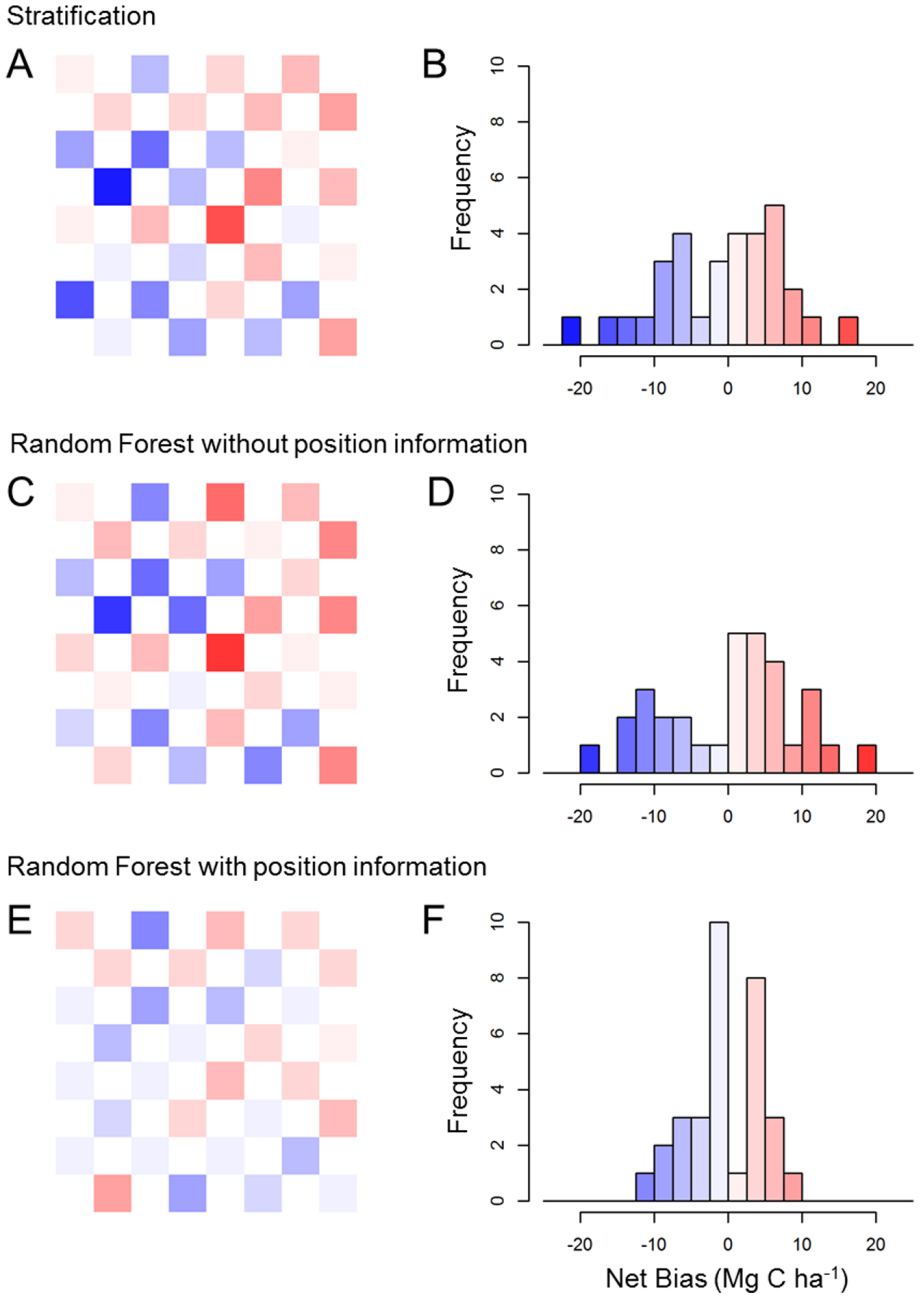
Spatial performance of each modeling approach within 36 validation regions. The net bias for the model was determined against the aircraft-based observed validation data (i.e., 1-ha CAO aircraft samples shown in black in figure 1). The color-scale is defined within the histograms, and is constant in all panels.

We examined the net bias in each of the 36 validation cells separately by summing all residuals between predicted ACD and aircraft-observed ACD within each cell. In doing so, we found that Random Forest with position information out-performed both Stratification and Random Forest without position information in most cells (Figure 7). Notably, only one of 36 cells exhibited an absolute net bias greater than 10 Mg C ha^−1^ when using Random Forest with position information, and the overall distribution of bias among validation cells exhibited greater kurtosis (i.e., the distribution is more peaked around zero; Figure 7).

**Figure 8:**
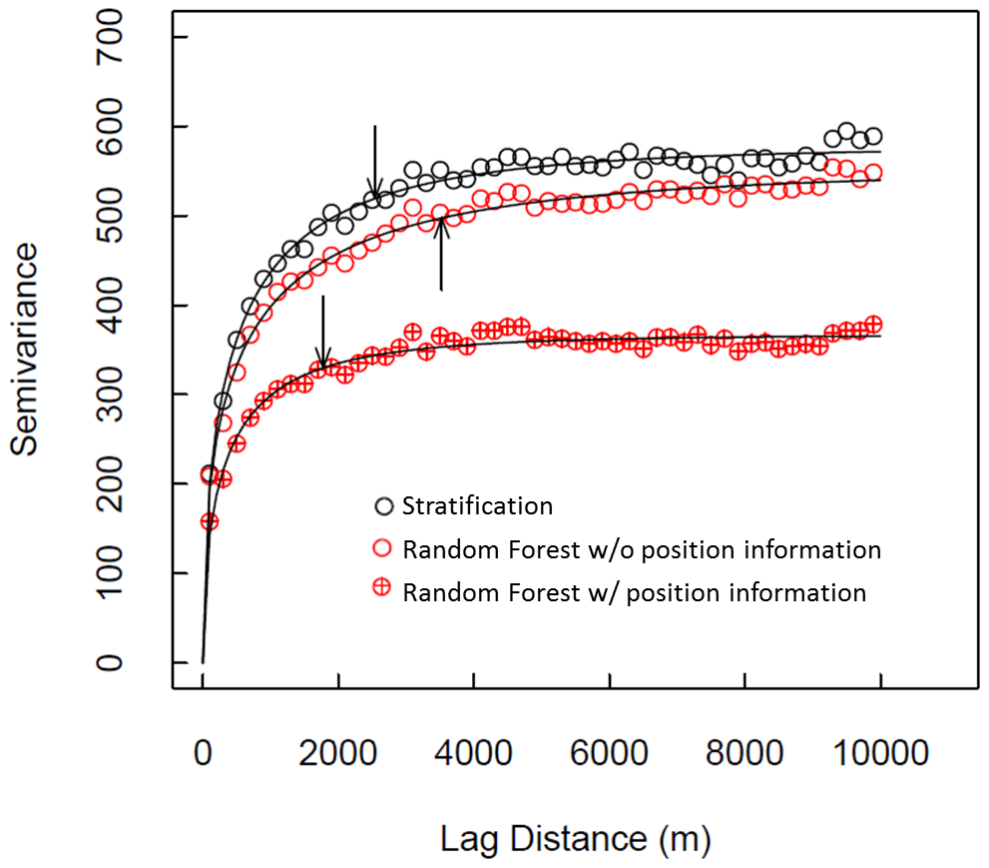
Variograms of residuals from three model predictions of carbon stock in the focal area: Stratification (black circles), Random Forest without position information (red open circles), and Random Forest with position information (red circles with target). Fitted curves are weibull functions, and the arrows indicate a y-axis value of 90% of the fitted asymptote (i.e., the “a” parameter in the weibull function).

We found that the residuals of all three models were positively spatially autocorrelated; i.e., errors were spatially clustered (Figure 8). Moran's I was highly significant in all cases (*P*≪0.0001): Random Forest without position information exhibited the highest Moran's I (0.1466), followed by stratification (0.1155), and Random Forest with position information (0.1153).

Finally, we detected over-fitting by Random Forest; both with and without position information, Random Forest's internal “out-of-bag” percent variation explained was 29% greater than what the percentages determined using the 36 validation cells.

## Discussion

Monitoring, reporting and verification is a critical part of any possible tropical forest carbon accounting system [11], [44]. But from individual landowners to entire nations, each jurisdictional entity must be able to determine not just how much carbon their land holds, but where that carbon is located. Ultimately, this means a spatially explicit, hectare-by-hectare capability is needed. We show here that Random Forest machine learning—if carefully implemented—can be a powerful spatial modeling tool to aid tropical forest carbon monitoring.

We evaluated two options for Random Forest, in the first case using a suite of input variables but lacking information on geographic position. This model performed similarly to the more traditional approach of stratification (sensu [7]), but it had some drawbacks that suggest high risk: model fit to validation data was somewhat better than stratification (i.e., modest decrease in RMSE and increase in adjusted r^2^), but a downside was increased spatial autocorrelation among the model residuals, as measured by a 27% increase in Moran's I when using Random Forest.

However, the second option, which included the same suite of input variables as well as four position parameters, produced much more accurate results with validation data compared to the stratification approach, and without increasing spatial autocorrelation in the residuals. Side-by-side comparison of the carbon maps resulting from the three techniques provides some insight into the improvement in model predictions when pixel position is included (Figure 5). Within the Pacaya-Samiria swampland in the focal area, for example, a band of floodplain forests several kilometers wide follows the Ucayali River (annotation 1 in Figure 5); these forests have high carbon stocks in LiDAR data, but occupy a very low-lying area relative to the rest of the focal area, and they appear to have common elevation, slope, and greenness to lower-carbon swamps and marshes elsewhere in the swampland. Similarly, context-dependent patterns appear to influence carbon stocks in the southwest portion of the focal area (annotation 2 in Figure 5). Here, high elevation forested valleys have relatively high predicted carbon stocks in all three models, but nearby east-facing mountain slopes maintain high greenness yet exhibit lower carbon stocks in LiDAR data; these areas have much lower predicted carbon stocks when position information is included in the Random Forest model. Ecologically, the results suggest that relative rather than absolute elevation may be more predictive of localized variation in carbon stocks in many instances throughout the region.

Our results suggest that the inclusion of position information is helpful—and potentially critical—to advancing Random Forest as an upscaling and modeling approach for tropical forest carbon mapping. With position information as model input variables, we suggest that Random Forest is better able to account for the context in carbon stock patterns—i.e., spatial autocorrelation of observed carbon stocks in LiDAR data—increasing its predictive accuracy in unseen data. To our knowledge, although Random Forest has been used in several ecological and spatial studies to date, position information has yet to be included in any predictive model of carbon or other ecological parameter [4], [16], [22], [23]. Our results suggest that future efforts should make better use of position information in order to improve predictive power and possibly better account for spatial autocorrelation. In principle, including position information in the stratification approach might lead to a similar improvement in performance. However, unlike Random Forest, the stratification approach lacks an algorithm to deconstruct which position information that is relevant to ACD patterns and which is not. Given that 8,035 classes were created from the intersection of the limited variable set considered in this case, the rapidly increasing number of classes may quickly make the exercise intractable.

Does Random Forest over fit to spatial data? Our results suggest that it does. Random Forest leaves out portions of the input data (called “out-of-bag”) to evaluate its prediction and this approach is theoretically less prone to over fitting to training data compared to other machine learning algorithms [22]. But, in implementing both instances of Random Forest here, the “out-of-bag” predictive power generated by the model was 29% higher than what we determined for validation areas never included at any stage. Ultimately, while over-fitting is not desirable, Random Forest with position information still produced the best results in unobserved areas as assessed by validation data (Figure 7). This suggests that care must be taken when using Random Forest with spatial data and that the internal “out-of-bag” feature appears not to be robust to spatial data.

Our mapping predictions exhibited spatial autocorrelation of errors, as has been the case with most other regional-, jurisdictional- and national-scale carbon mapping efforts. While including position information as input variables in Random Forest did reduce spatial autocorrelation, it did not eliminate it (i.e., Moran's I declined from 0.1466 to 0.1153, which was a difference outside the standard deviation of each Moran's I estimate). With position information, Random Forest exhibited no statistical difference in spatial autocorrelation from the stratification approach (at a Moran's I of 0.1155), yet produced a major improvement in model performance in terms of RMSE and r^2^ in validation data. Although the spatial autocorrelation of errors is undesirable, Random Forest with position information provided an improvement over stratification, and maintained the same spatial autocorrelation as with stratification.

Machine-learning algorithms have the potential to substantially improve spatial modeling of carbon stocks in tropical forests and possibly other ecosystems. Although some drawbacks remain unresolved—namely over-fitting and spatial autocorrelation of model errors—Random Forest may provide a viable pathway to improve large-area modeling of carbon stocks over existing methods such as stratification. This is particularly true in large-scale, high-resolution modeling exercises that are currently intractable when using parametric statistical approaches such as simultaneous autoregressive modeling due to computational limitations (e.g., SAR [37], [45]). We emphasize that our modeling outcomes greatly benefited from an unprecedentedly high density of airborne LiDAR data over a large geographic region, and this suggests that high data density may be critical moving forward. Further, testing Random Forest against other modeling approaches beyond stratification (e.g., k-nearest neighbor, maximum entropy) is also critical to determine its ultimate utility in carbon mapping.
